# Dual targeting of PDPN and GD2 enhances CAR T cell efficacy against glioblastoma and promotes durable tumor control

**DOI:** 10.1016/j.omton.2026.201318

**Published:** 2026-09-04

**Authors:** Julian Hübner, Thomas Nerreter

**Affiliations:** 1 Chair of Cellular Immunotherapy, Department of Medicine II, University Hospital Würzburg, Würzburg, Germany

## Main text

Glioblastoma (GBM) is the most common primary malignant brain tumor in adults and remains almost universally fatal despite the current standard of care, which comprises maximal surgical resection followed by concomitant and adjuvant chemoradiotherapy as well as tumor-treating fields.^1^^,^^2^^,^^3^^,^^4^ Effective therapeutic breakthroughs remain elusive, underscoring the urgent need for novel experimental treatment strategies. One of the most successful immunotherapeutic approaches in hematologic malignancies is chimeric antigen receptor (CAR) T cell therapy, which has achieved remarkable response rates and durable remissions across several cancer types.^5^ In contrast, comparable success has not yet been achieved in solid tumors, and CAR T cell therapy has therefore not advanced to clinical routine in these malignancies. This can be attributed to several factors, including the lack of tumor-specific target antigens, pronounced antigen heterogeneity, an immunosuppressive tumor microenvironment, and limited or impaired trafficking of CAR T cells to the tumor site, as is particularly evident in GBM.^6^^,^^7^ In our recent publication in *Journal for ImmunoTherapy of Cancer*, we therefore sought to identify robust, broadly expressed tumor-associated antigens and develop a locally administered dual-targeting CAR T cell strategy capable of overcoming the pronounced heterogeneity of GBM.^8^

To achieve this, we performed a comprehensive antigen discovery campaign combining flow cytometric analysis of freshly resected GBM tissue with immunohistochemical analysis of matched primary and recurrent formalin-fixed paraffin-embedded specimens. These analyses identified podoplanin (PDPN) and the ganglioside GD2 as consistently expressed target antigens in GBM, both exhibiting increased expression in recurrent tumors. Furthermore, analyses of The Cancer Genome Atlas GBM dataset, together with qPCR analyses, demonstrated that PDPN expression has a strong inverse correlation with patient survival while remaining comparatively low in healthy tissues. Collectively, these findings established PDPN and GD2 as attractive candidates for a novel dual-targeting CAR T cell approach.

We subsequently generated PDPN- and GD2-specific CAR T cells and confirmed their individual therapeutic efficacy across a panel of GBM cell lines (GCLs). These experiments revealed complementary antitumor activity, as both CAR T cell products displayed overlapping yet distinct cytotoxicity profiles and interferon gamma (IFN-γ) secretion patterns across the GCL panel. Building on these observations, we developed several dual-targeting CAR T cell formats, including co-administration of separately manufactured PDPN- and GD2-directed CAR T cells at a 1:1 ratio, co-transduction, tandem CARs, and bicistronic vectors. While all approaches generated functional CAR T cell products, systematic comparison in co-culture assays demonstrated that co-administration of individually manufactured PDPN- and GD2-CAR T cells, referred to as “blend” in our study, provided the most favorable balance of cytotoxicity, proliferation, and cytokine production. Moreover, this modular approach avoids the increased engineering complexity associated with multi-specific CAR constructs while maintaining efficient CAR T cell manufacturing and functionality.

To evaluate the functional activity and potential therapeutic benefit of the PDPN/GD2-CAR T cell blend in a more heterogeneous setting, we established co-culture models using patient-derived organoids (PDOs). Unlike established cell lines, which only partially recapitulate the characteristics of the parental tumor following prolonged two-dimensional culture, PDOs generated directly from freshly resected GBM tissue preserve key molecular and cellular features of the original tumor, even after extended culture, cryopreservation, and thawing.^9^ Consequently, PDOs provided a challenging three-dimensional model that more faithfully reflects GBM heterogeneity and allowed us to assess CAR T cell infiltration, cytotoxicity, and cytokine production in a clinically relevant context.

Within this model, anti-tumor responses were assessed after 20 h of co-culture to preserve tissue integrity while enabling immunofluorescence imaging. We observed robust CAR T cell infiltration into PDOs, together with antigen-specific induction of apoptosis, as demonstrated by cleaved caspase-3 staining. In parallel, apoptosis was quantified using the Caspase-Glo 3/7 3D assay, while cytokine secretion was analyzed using LEGENDplex assays. Although all CAR T cell products effectively targeted PDOs, dual targeting with the PDPN/GD2-CAR T cell blend resulted in substantially greater antitumor activity than either monovalent CAR T cell product. This was reflected by significantly enhanced induction of apoptosis, together with favorable release of effector molecules such as IFN-γ, granzyme B, and Fas ligand.

To complete our preclinical evaluation, we investigated whether dual targeting with the PDPN/GD2-CAR T cell blend can also confer superior efficacy *in vivo*. We established a heterogeneous orthotopic NSG mouse model by stereotactically implanting U87 ffluc/GFP^+^ cells into the right caudate nucleus on day 0. This model proved particularly well suited for our studies, as U87 cells exhibit heterogeneous expression of PDPN and GD2 *in vitro*, giving rise to distinct populations expressing either antigen alone, both antigens, or neither antigen. Furthermore, antigen expression remained highly plastic following intracranial implantation, thereby providing an ideal experimental setting to evaluate the advantages of dual-antigen targeting.

Following confirmation of tumor engraftment by bioluminescence imaging, CAR T cells were administered on day 7. Based on previous reports demonstrating superior efficacy of locoregional compared with intravenous delivery,^10^ CAR T cells were injected into the lateral ventricle of the opposing left hemisphere, thereby introducing an additional challenge for CAR T cell migration toward the tumor. Monovalent PDPN- and GD2-directed CAR T cells induced marked tumor regression; however, tumors in both treatment groups ultimately relapsed. In contrast, the dual-targeting CAR T cell blend mediated sustained tumor control, producing curative responses in the majority of animals and leaving no detectable residual tumor at long-term follow-up, as confirmed by flow cytometric analysis.

In summary, our study presents a novel dual-targeting CAR T cell strategy for the treatment of heterogeneous glioblastoma. By simultaneously targeting PDPN and GD2, this approach addresses one of the principal obstacles limiting CAR T cell therapy in GBM, antigen heterogeneity, and demonstrates substantial therapeutic potential in both advanced *in vitro* models and orthotopic *in vivo* studies. These findings provide a strong preclinical rationale for the further development of dual-targeting CAR T cell therapies as a promising alternative to conventional monovalent CAR T cell approaches for patients with GBM.

## Declaration of interests

T.N. is listed as inventor on patent applications and granted patents related to CAR T cell technologies that have been filed by the University of Würzburg, Würzburg, Germany, and that have been, in part, licensed by industry. T.N. is employed at T-CURX GmbH, Würzburg, Germany.
